# Clinical Global Impression-severity score as a reliable measure for routine evaluation of remission in schizophrenia and schizoaffective disorders

**DOI:** 10.1186/s12991-015-0042-6

**Published:** 2015-02-13

**Authors:** Federica Pinna, Luca Deriu, Enrica Diana, Valeria Perra, Rachele Pisu Randaccio, Lucia Sanna, Massimo Tusconi, Bernardo Carpiniello, Cagliari Recovery Study Group

**Affiliations:** Department of Public Health, Clinical and Molecular Medicine-Unit of Psychiatry, University of Cagliari, Via Liguria 13, 09127 Cagliari, Italy

## Abstract

**Aims:**

This study aimed to compare the performance of Positive and Negative Syndrome Scale (PANSS) symptom severity criteria established by the Remission in Schizophrenia Working Group (RSWG) with criteria based on Clinical Global Impression (CGI) severity score. The 6-month duration criterion was not taken into consideration.

**Methods:**

A convenience sample of 112 chronic psychotic outpatients was examined. Symptomatic remission was evaluated according to RSWG severity criterion and to a severity criterion indicated by the overall score obtained at CGI-Schizophrenia (CGI-SCH) rating scale (≤3) (CGI-S).

**Results:**

Clinical remission rates of 50% and 49.1%, respectively, were given by RSWG and CGI-S, with a significant level of agreement between the two criteria in identifying remitted and non-remitted cases. Mean scores at CGI-SCH and PANSS scales were significantly higher among remitters, independent of the remission criteria adopted. Measures of cognitive functioning were largely independent of clinical remission evaluated according to both RSWG and CGI-S. When applying RSWG and CGI-S criteria, the rates of overall good functioning yielded by Personal and Social Performance scale (PSP) were 32.1% and 32.7%, respectively, while the mean scores at PSP scale differed significantly between remitted and non-remitted patients, independent of criteria adopted. The proportion of patients judged to be in a state of well-being on Social Well-Being Under Neuroleptics-Short Version scale (SWN-K) were, respectively, 66.1% and 74.5% among remitters according to RSWG and CGI-S; the mean scores at the SWN scale were significantly higher only among remitters according to CGI-S criteria.

**Conclusions:**

CGI severity criteria may represent a valid and user-friendly alternative for use in identifying patients in remission, particularly in routine clinical practice.

## Introduction

Clinical remission [1] is viewed nowadays as an achievable goal in the treatment of schizophrenia and related disorders, with sustained remission representing one of the main steps involved in achieving recovery [2]. The lack of a univocal method for assessing remission has long represented a major methodological problem, particularly due to the largely different set of criteria used in clinical research, relating to symptom domains included, type of rating scales adopted and time-points considered in defining remission [3]. A significant step forward was made following publication of the Remission in Schizophrenia Working Group criteria (RSWGcr) [4]. These criteria establish clinical remission based on a symptom severity criterion comprising several crucial items from some of the most relevant rating scales used in schizophrenia (Positive and Negative Syndrome Scale (PANSS), Brief Psychiatric Rating Scale (BPRS), SANS, SAPS); with particular regard to PANSS, eight items were identified as the most diagnostic-specific for schizophrenia (i.e. they accounted for core symptoms necessary for a diagnosis of schizophrenia according to DSM-IV-TR), and a duration criterion of 6 months was introduced. As PANSS scale provides ratings that investigate not only symptom severity per se but also functional impairment, a score of “mild” or better (i.e. 3 points or less) at all eight “core” symptoms was considered sufficiently representative of a level of impairment consistent with symptomatic remission of the disorder [4]. The validity and utility of the cross-sectional symptom and time criteria were evaluated [5,6], and both were found to be clinically meaningful in evaluating remission [7,8]. However, symptom severity was the only criterion adopted in the majority of studies [9], in spite of criticism that this may inflate rates of remission detected. RSWG criteria have proved to be conceptually viable and easy to use both in clinical trials and clinical practice [10]; however, as the use of PANSS or BPRS rating scales in routine practice is time-consuming and linked to the knowledge of specific skills by clinicians, the need for simpler and easier means of evaluating remission is highlighted. Accordingly, Leucht et al. [11] proposed use of the Clinical Global Impression Scale in pragmatic trials, while Masand et al. [3] demonstrated that Clinical Global Impression (CGI)-Improvement score is a valid proxy measure for remission in schizophrenia. Starting from these premises, the present study was designed to evaluate the efficacy of a Clinical Global Impression-Schizophrenia (CGI-SCH) Severity score equal to or lower than 3 as a measure of remission in comparison to PANSS-based RSWG criteria [4]. In particular, clinical remission was evaluated according to functional status, quality of life and cognitive outcomes [12].

## Materials and methods

### Sample

In the context of a prospective study [13], a convenience sample comprising all chronic outpatients with a diagnosis of schizophrenia or schizoaffective disorder according to DSM-IV-TR attending a university community mental health centre (CMHC) in the year 2010 was recruited consecutively. Patients with other comorbid psychiatric disorders, including substance use disorders, and/or somatic disorders were also included in the study. Patients affected by comorbid mental retardation or organic brain diseases were excluded from the study. Standard care routinely available in Italian CMHCs was provided (clinical monitoring at least on a monthly basis; pharmacological treatment; home care when required, psychosocial and rehabilitation interventions tailored to patient’s needs).

### Ratings

The study was approved by the Ethics Committee of Local Health Unit n.8, Cagliari and was performed in compliance with national laws. Evaluation was performed by residents in psychiatry using a set of standardized methods, after adequate training in use of all instruments adopted. Clinical ratings for each single patient were generally carried out by different residents whenever possible. Patients were interviewed by means of the SCID-I [14] and SCID-II [15], after providing informed consent. Interviews were conducted by residents in psychiatry trained in use of the instruments; inter-rater reliability, assessed using Cohen’s *K* before the study, was higher than 0.80. Personal and social data and clinical history were collected through a structured interview purpose-developed for the study and from standardized records routinely used in the centre and based on procedures suggested by the Association for Methodology and Documentation in Psychiatry (AMDP) [16], as described in previous studies [17,18]. Symptom severity was evaluated using PANSS [19]. Interviews were conducted by residents in psychiatry trained in use of the instrument; inter-rater reliability of PANSS evaluations in terms of intraclass correlation coefficient (ICC) for PANSS total score ranged from 0.65 to 0.95. RSWG criteria [4] based on ratings at eight focal symptoms in positive, negative and general psychopathology subscales of PANSS (P1, P2, P3, N1, N4, N6, G5, G9) were applied for clinical remission. Patients were judged to be in clinical remission according to a severity criterion (scores obtained at each item ≤3 points, indicating mild severity of symptoms). Due to the cross-sectional nature of the study, clinical remission was evaluated taking into account the severity criterion alone, excluding the duration criterion (remission maintained for 6 months). Clinical status was also evaluated by the CGI-SCH [20] by residents in psychiatry trained in use of the instrument. Inter-rater reliability of CGI-SCH evaluations in terms of ICC for CGI-SCH total score ranged from 0.69 to 0.96; the criterion suggested by Leucht et al. [11], indicating a severity score equal to or lower than 3 (slightly ill) to indicate remission, was used. When applying PANSS, remission was evaluated excluding the 6-month criterion even when CGI-SCH was evaluated. Cognitive functioning was evaluated by means of the Brief Assessment of Cognition in Schizophrenia scale (BACS) [21] for which a gender/age/education-adjusted score and thus an equivalent score were calculated [22]. Mini Mental State Examination (MMSE) test [23] was administered to calculate an age/education-adjusted score [24]. Functioning was evaluated by Personal and Social Performance scale (PSP) [25], to assess social functioning of patients in four main areas: socially useful activities, personal and social relationships, self-care and disturbing/aggressive behaviours. A comprehensive overall score ranging from 1 (maximum dysfunction) to 100 (maximum functioning) was attributed, based on score obtained at each single area. A total score exceeding 70 indicates a condition of “functional remission”, i.e. an overall good functioning. Subjective well-being was evaluated by means of Subjective Well-Being Under Neuroleptics-Short Version (SWN-K) [26], a self-administered 20-item rating scale aimed at assessing the psychological and physical well-being of patients treated with neuroleptics. An overall score equal to or higher than 80 indicates a state of subjective well-being.

### Statistical analysis

Categorical data were analyzed using Pearson’s χ^2^ test or Fisher’s exact test; continuous variables were assessed by means of Student’s *t* test for independent samples. The magnitude of differences in mean scores obtained at different rating scales used in the study was calculated by means of Cohen’s *d*. To evaluate differences in remission rates observed according to the different proposed criteria, McNemar’s test for matched pairs of subjects was used. Phi coefficient was used to evaluate correlations between rates of remission evaluated by means of RSWG criteria and CGI-Severity criteria Data analyses were performed using SPSS 19.0. Level of significance was set at a *p* value ≤0.05 for two-tailed hypothesis.

## Results

### Baseline characteristics

The sample comprised 112 patients, 80 (71.4%) males and 32 (28.6%) females and 46 (41.1%) schizophrenic and 66 (58.9%) schizoaffectives; 35 patients (31.3%) were affected by comorbid medical illnesses and 32 (28.5%) by comorbid substance abuse disorders; the mean age was 43.5 ± 9.42 years (range 25–68); mean years of education 10.84 ± 3.9 (range 4–24); 97 subjects (86.6%) were single; 83 (74.1%) unemployed.

### Clinical remission

A similar proportion of remitted patients was obtained using both RSWG criteria (*n* = 56, 50%) and CGI-Severity criteria (*n* = 55, 49.1%) (*p* = 1.000). According to RSGW, 43.5% of schizophrenics (*n* = 20) and 54.5% of schizoaffectives (*n* = 36) were “remitted” (*p* = 0,249), while the proportion of “remitted” patients according to CGI criteria was 52% (*N* = 24) and 47% (*n* = 31), respectively, for patients affected by schizophrenia and schizoaffective disorder (*p* = 0.588). From the total sample, 37.5% (*n* = 42) were found to be in remission and 38.4% (*n* = 43) non-remitted according to both RSWG and CGI-S criteria (Phi = 0.518, *p* < .0001). No significant differences in remission rates, using either RSWG or CGI criteria, were found between patients both with and without comorbid medical illnesses or substance use disorders. Remitted patients featured several significantly different characteristics, independent of remission criteria adopted, as shown in Table 1. Mean scores at CGI-SCH and PANSS were all significantly higher among non-remitted patients yielding effects of medium (Cohen’s *d* 0.5–1) or large (Cohen’s *d* > 1) magnitude for the majority of scales, independent of remission criteria (Table 2). Similar results were found on considering schizophrenic and schizoaffective patients separately. Indeed, mean scores at CGI-SCH and PANSS were significantly higher (all *p* values ranging between <0.002 and <0.0001) among non-remitted schizophrenic and schizoaffective patients, independent of remission criteria used. The only exception was represented by CGI-SCH-Cognitive symptoms scale, revealing no significant differences in mean scores obtained by both groups, independent of remission criteria adopted.

**Table 1:** **Sociodemographic characteristics of remitted and non-remitted patients according to different criteria**

**Items**	**Criteria of remission**	**Remitted**	**Non-remitted**	**Statistics (df)**
Education (years) (means ± SD)	SWGC	11.55 (4.16)	10.13 (3.43)	*t*(110) = 1.981 *p* = .050
	CGI-S	11.55 (4.01)	10.16 (3.62)	*t*(110) = 1.922 *p* < .057
Occupation (unemployed) *N* (%)	SWGC	36 (64.3%)	47 (83.9%)	Chisq(1) = 9.775 *p* < .0001
	CGI-S	35 (63.3%)	48 (84.2%)	Chisq(1) = 6.175 *p* = .013
Course of illness (continuous + episodic with residual symptoms) *N* (%)	SWGC	39 (69.6%)	50 (89.3%)	Chisq(1) = 9.560, *p* = .008
	CGI-S	36 (65.5%)	53 (92.9%)	Chisq(1) = 23.343 *p* < .0001
Duration of illness (months) (means ± SD)	SWGC	163.68 (100.01)	227.48 (112.58)	*t*(110) = −3.171, *p* = .002
	CGI-S	173.04 (106.72)	217.33 (111.11)	*t*(110) = −2.151, *p* = .034

*SWGC* Schizophrenia Working Group Severity Criterion, *CGI* Clinical Global Impression Severity Criterion.

**Table 2:** **Mean scores ± sd at clinical scales of remitted and non-remitted patients according to different criteria**

**Items**	**Criteria of remission**	**Remitted**	**Non-remitted**	**Statistics (df)/** ***p*** **Cohen’s** ***d***
CGI-S positive symptoms	SWGC	1.60 (0.95)	2.95 (1.42)	*t*(110) = −5.853, *p* < .0001/−1.17
	CGI-S	1.49 (0.84)	3.07 (1.37)	*t*(110) = −7.316, *p* < .0001/−1.39
CGI-S negative symptoms	SWGC	1.78 (0.91)	3.36 (1.27)	*t*(110) = −7.478, *p* < .0001/−1.43
	CGI-S	1.80 (0.87)	3.39 (1.36)	*t*(110) = −7.326, *p* < .0001/−1.39
CGI-S depressive symptoms	SWGC	1.71 (0.85)	2.36 (1.31)	*t*(110) = −3.076, *p* = .003/−0.58
	CGI-S	1.53 (0.716)	2.61 (1.40)	*t*(110) = −5.148, *p* = .0001/−0.98
CGI-S cognitive symptoms	SWGC	1.84 (1.03)	3.18 (1.20)	*t*(110) = −6.298, *p* < .0001/−1.19
	CGI-S	1.85 (1.08)	3.14 (1.88)	t(110) = −5.999, *p* < .0001/−1.14
CGI-S overall severity	SWGC	2.45 (0.95)	3.82 (0.76)	*t*(110) = −8.309, *p* < .0001/−1.59
	CGI-S	2.24 (0.66)	4.07 (0.65)	*t*(110) = −14.74, *p* < .0001/−2.81
PANSS positive scale	SWGC	8.96 (2.09)	14.39 (4.35)	*t*(110) = −8.417, *p* < .0001/−1.59
	CGI-S	9.55 (2.70)	13.74 (4.67)	*t*(110) = −5.788, *p* < .0001/−1.10
PANSS negative scale	SWGC	10.57 (3.65)	18.70 (5.85)	*t*(110) = −8.803, *p* < .0001/−1.66
	CGI-S	10.96 (3.68)	18.18 (6.39)	*t*(110) = −7.287, *p* < .0001/−1.39
PANSS general psychopathology	SWGC	21.98 (4.87)	32.68 (7.48)	*t*(110) = −8.964, *p* < .0001/−1.69
	CGI-S	22.51 (4.83)	31.98 (8.25)	*t*(110) = −7.378, *p* < .0001/−1.41
PANSS total scale	SWGC	41.52 (7.92)	65.77 (13.87)	*t*(110) = −11.35, *p* < .0001/−1.69
	CGI-S	43.02 (8.82)	63.89 (15.87)	*t*(110) = −8.559, *p* < .0001/−1.63

*SWGC* Schizophrenia Working Group Severity Criterion, *CGI* Clinical Global Impression Severity Criterion.

### Cognitive functioning

Both RSWG and CGI-S criteria detected very similar mean scores for cognitive functioning (Table 3) between remitters and non-remitters, with the sole exception of MMSE, featuring higher scores among remitters for RSWG criteria, and BACS-Digit sequencing task, with significantly higher scores among remitters using both RSWG and CGI-S criteria. Considering separately the two diagnostic subgroups, significantly higher scores were found for Fluency/category instances (*p* = 0.003) and Verbal Fluency/Controlled Oral Words association test (*p* = 0.010) among schizophrenics judged as remitted according to RSWG criteria and for Verbal Fluency/Controlled Oral Words association test (p = 0.045) among schizophrenics judged as remitted according to CGI criteria. In schizoaffective patients, significantly higher mean scores were found only for Digit sequencing task, using both RSWG criteria (*p* = 0.011) and CGI criteria (*p* < 0.0001).

**Table 3:** **Mean score ± sd at neuropsychological tests in remitted and non-remitted patients according to different criteria**

**Items**	**Criteria of remission**	**Remitted**	**Non-remitted**	**Statistics (df)** ***/p*** **Cohen’s** ***d***
MMSE total score	SWGC	26.80 (2.71)	25.02 (4.42)	*t*(110) = 2.576, *p* = .011/0.485
	CGI-S	26.40 (3.20)	25.44 (4.21)	*t*(110) = 1.358, *p* = .177/NA
BACS list learning	SWGC	9.31 (4.77)	9.92 (4.59)	*t*(99) = −.652, *p* = 0.516/NA
	CGI-S	9.51 (4.78)	9.70 (4.53)	*t*(101) = .199, *p* = 0.843/NA
BACS Digit sequencing task	SWGC	14.75 (6.06)	11.26 (6.19)	*t*(99) = 2.86, *p* = .005/0.571
	CGI-S	15.37 (5.56)	10.34 (6.13)	*t*(101) = 4.363, *p* < .0001/0.868
BACS Verbal Fluency/category instances	SWGC	9.89 (4.98)	8.10 (4.96)	*t*(99) = 1.801, *p* = .075/NA
	CGI-S	9.63 (5.07)	8.25 (4.84)	*t*(101) = 1.405, *p* = .163/NA
BACS Verbal Fluency Controlled Oral Words ass.test	SWGC	15.05 (3.89)	14.50 (5.49)	*t*(99) = 0.569, *p* = .571/NA
	CGI-S	15.47 (4.61)	13.98 (4.75)	*t*(101) = 1.613, *p* = .110/NA
BACS Symbol coding	SWGC	32.60 (13.64)	28.60 (12.29)	*t*(99) = 1.549, *p* = 0.125/NA
	CGI-S	31.84 (13.03)	28.71 (13.15)	*t*(101) = 1.214, *p* = .228/NA
BACS Tower of London	SWGC	11.38 (5.98)	9.5 (6.53)	*t*(99) = 1.445, *p* = 0.152/NA
	CGI-S	10.96 (6.20)	9.77 (6.27)	*t*(101) = .969, *p* = .228/NA

*SWGC* Schizophrenia Working Group Severity Criterion, *CGI* Clinical Global Impression Severity Criterion, *NA* not assessed in the absence of significant difference.

### Functioning

Twenty-three patients (20.5%), particularly 13% of schizophrenics (*n* = 6) and 25.8% of schizoaffectives (*n* = 17) (*p* = 0.101) were found to be in functional remission*.* The proportion of “functionally remitted” patients who were clinically remitted according to RSWG criteria was 15% (*n* = 3) for schizophrenics (*p* = 0.730) and 41.7% for schizoaffectives (*n* = 15) (*p* = 0.001); CGI criteria indicated proportions of 20.8% (*n* = 5) and 41.9% (*n* = 13), respectively, for schizophrenics (*p* = 0.101) and schizoaffectives (*p* = 0.05). Overall, the rates of “functionally” remitted patients judged also to be in clinical remission according to RSWG and CGI-S criteria were 32.1% (*n* = 18) and 32.7% (*n* = 18), respectively. On the contrary, rates of non-“functionally” remitted patients also deemed not to be in clinical remission were, respectively, 91.2% (*n* = 52) and 91.1% (*n* = 51). Phi coefficients were 0.287 (*p* = .002) and 0.296 (*p* = .002), respectively, using RSWG and CGI-S criteria. Significantly higher mean scores at PSP subscales and significantly lower ones at PSP total scale were found among non-remitters, indicating poorer functioning (Table 4), independent of remission criteria adopted. The sole exception was observed for PSP self care and PSP aggressive-disturbing behaviour, where differences in mean scores, although lower, did not differ significantly when using CGI-S criteria to evaluate remission; a similar magnitude of effect sizes was revealed between the clinically remitted according to RSWG and CGI-S criteria. Considering the two groups of patients separately, we found that for schizophrenics, mean scores at PSP-socially useful activities subscale were significantly higher among patients judged to be in clinical remission using both SWGC and CGI criteria (respectively, *p* = 0.012 and *p* = 0.002). Mean scores obtained at PSP-personal relationships and PSP-total scores were significantly higher only among clinical “remitters” according to CGI criteria (*p* = 0.002 and *p* < 0.0001, respectively); no significant differences were found in mean scores at PSP-aggressive/disturbing behaviours and PSP-Self-care subscales, independent of the criterion adopted for evaluating clinical remission. Significantly higher scores were obtained for clinically remitted schizoaffective patients according to RSWG criteria at all PSP subscales and PSP-total score (all values between *p* < 0.005 and *p* < 0.0001). Applying CGI criteria, mean scores were significantly higher at PSP-Socially useful and Personal relationships subscales and PSP-Total score (all values *p* < 0.0001), while no significant difference was found at PSP-aggressive/disturbing behaviour and PSP-Self-care subscales.

**Table 4:** **Results at PSP scale in remitted and non-remitted patients according to different criteria**

**Items**	**Criteria of remission**	**Remitted**	**Non-remitted**	**Statistics(df)/Cohen’s** ***d***
PSP-activities (means ± SD)	SWGC	1.88 (1.27)	3.20 (1.21)	*t*(110) = −5.64, *p* < .0001/−1.06
	CGI-S	1.76 (1.12)	3.28 (1.23)	*t*(110) = −6.79, *p* < .0001/−1.29
PSP-social rel (means ± SD)	SWGC	2.02 (1.15)	2.86 (1.15)	*t*(110) = −5.64, *p* < .0001/−0.73
	CGI-S	1.91 (1.04)	2.95 (1.71)	*t*(110) = −4.95, *p* < .0001/−0.94
PSP-self care (means ± SD)	SWGC	0.34 (0.69)	0.80 (1.16)	*t*(110) = −2.56, *p* < .012/−0.48
	CGI-S	0.45 (0.86)	0.68 (.1,09)	*t*(110) = −1.24, *p* = .218/NA
PSP-aggressive and disturbing behaviour (means ± SD)	SWGC	0.14 (0.44)	0.50 (0.81)	*t*(110) = −2.89, *p* < .005/−0.55
	CGI-S	0.24 (0.64)	0.40 (0.70)	*t*(110) = −1.32, *p* = .191/NA
PSP total score (means ± SD)	SWGC	62.27 (13.65)	50.38 (14.79)	*t*(110) = 4.43, *p* < .0001/0.83
	CGI-S	64.84 (11.49)	48.11 (14.21)	*t*(110) = 6.84, *p* < .0001/1.30

*SWGC* Schizophrenia Working Group Severity Criterion, *CGI* Clinical Global Impression Severity Criterion, *NA* not assessed in the absence of significant difference.

### Subjective well-being

Seventy-one patients (63.4%) were found to be in a state of “well-being”, in particular 69.6% of schizophrenics (*n* = 32) and 59.1% of schizoaffectives (*n* = 39) (*p* = 0.258). The proportion of subjects in a state of subjective well-being among those clinically remitted according to RSWG criteria was 70% (*n* = 14) for schizophrenics (*p* = 0.955), and 63.9% for schizoaffectives (*n* = 23) (*p* = 0.385); according to CGI criteria, the proportions were 70.8% (*n* = 17) and 77.4% (*n* = 24), respectively, for schizophrenics (*p* = 0.845) and schizoaffectives (*p* = 0.04). Overall, the rates of subjects who were “clinically remitted” and in a state of “well-being” were 66.1% (*n* = 37) and 74.5% (*n* = 41), respectively, using RSWG and CGI-S criteria. The rates of subjects who were “clinically non-remitted” and in a state of “absence of well-being” were 39.3% (*n* = 22) and 47.4% (*n* = 27), respectively. Phi coefficients were 0.056 (*p* = .556) and 0.227 (*p* = .016), respectively, using RSWG and CGI-S criteria. Mean scores were significantly higher at SWN subscales and total scale only among clinically remitted when using CGI-S criteria (Table 5), with effect sizes of a generally moderate magnitude. Considering the two groups of patients separately, no statistically significant differences were found in mean SWN total score and at all subscales between clinically remitted and non-remitted schizophrenic patients using RSWG criteria, while the only difference detected using CGI criteria was observed for the subscale “Mental functioning”, where remitted patients scored significantly higher (*p* = 0.014). Likewise, in schizoaffective patients, no statistically significant differences were detected at SWN total score and at all subscales between clinically remitted and non-remitted patients using RSWG criteria, while using CGI criteria, significantly higher scores (*p* values between <0.01 and <0.0001) were found at all SWN subscales and total score among remitted patients.

**Table 5:** **Results at SWN scale in remitted and non-remitted patients according to different criteria**

**Items**	**Criteria of remission**	**Remitted**	**Non-remitted**	**Statistics(df)/Cohen’s** ***d***
SWN-mental functions (means ± SD)	SWGC	16.98 (3.44)	15.79 (4.59)	*t*(110) = 1.56, *p* = .121/NA
	CGI-S	17.96 (3.39)	14.86 (4.12)	*t*(110) = 4.33, *p* < .0001/0.826
SWN-self control (means ± SD)	SWGC	17.59 (3.96)	16.21 (4.07)	*t*(110) = 1.81, *p* < .073/NA
	CGI-S	18.15 (3.69)	15.70 (4.06)	*t*(110) = 3.33, *p* = .001/0.634
SWN-physical functions (means ± SD)	SWGC	17.79 (3.53)	16.98 (3.96)	*t*(110) = 1.13, *p* = 260/NA
	CGI-S	18.35 (3.22)	16.46 (4.03)	*t*(110) = −2.74, *p* = .007/0.521
SWN-emotional control (means ± SD)	SWGC	16.71 (3.94)	16.91 (4.52)	*t*(110) = −.245, *p* = 807/NA
	CGI-S	17.98 (3.80)	5.68 (4.33)	*t*(110) = −2.97, *p* = 004/0.566
SWN-social activities (means ± sd)	SWGC	17.57 (3.15)	16.70 (4.03)	*t*/(110) = 1.281, *p* = .203/NA
	CGI-S	17.89 (3.40)	16.40 (3.71)	*t*(110) = −2.21, *p* = .029/0.421
SWN total score (means ± SD)	SWGC	86.64 (14.03)	82.59 (15.85)	*t*(110) = 1.433, *p* = .155/NA
	CGI-S	90.33 (13.31)	79.11 (14.64)	*t*(110) = 4.23, *p* < .0001/0.808

*SWGC* Schizophrenia Working Group Severity Criterion, *CGI* Clinical Global Impression Severity Criterion, *NA* not assessed in the absence of significant difference.

## Discussion

To our knowledge, this is the first study comparing remission evaluated by means of the Clinical Global Impression-Schizophrenia severity scale and RSWG criteria. Indeed, Masand et al. [3] used the CGI-Improvement score for this purpose, demonstrating that a score of 1 (very much improved) at week 4 of treatment correlated with the remission criteria developed by RSWG at study endpoint. In our sample, a very similar proportion of subjects, 50% and 49.1%, respectively, were found to be in clinical remission according to RSWG criteria [4] and CGI severity criteria, a result in line with other studies performed in different settings adopting mostly RSWG criteria [27-29]. Moreover, we found a highly significant correlation between the two systems in the identification of remitters and non-remitters; mean scores at clinical rating scales were significantly higher among remitters evaluated by means of both RSWG and CGI-S criteria, with a very similar magnitude of differences in mean scores between remitters and non-remitters using the two sets of criteria. No significant differences were found in mean scores at the majority of neurocognitive measures assessed between remitted and non-remitted patients, both using RSWG and CGI-S criteria. These results seem to support the hypothesis of a decline in cognitive functioning independent of clinical status, as shown recently by Shrivastava et al. [30] in a cohort of schizophrenic patients displaying a significant clinical improvement after 10 years of treatment. In our study, 23 patients (20.5%) were found to be in functional remission; approximately one third of clinically remitted subjects, according to both RSWG and CGI-S criteria, were found to be “functionally remitted”, with an extremely large and similar proportion of patients (approx. 91%) who were both clinically and functionally unremitted, irrespective of remission criteria adopted. This finding is in line with evidence reported of an incomplete recovery in schizophrenic disorders [1] and with the opinion generally shared by psychiatrists that patients affected by schizophrenia generally show impaired or very poor levels of functioning [31]. Furthermore, our data demonstrate that clinical remission is related to a similar degree of functional remission when evaluated by means of CGI-S and RSWG criteria. As expected from previous studies [27,28], mean PSP scores were unfailingly significantly higher among remitters than non-remitters. In our study, this finding was largely independent of remission criteria applied, with the same magnitude of differences in mean scores, thus providing further confirmation of the similar performances produced by these criteria. Further confirmation of the validity of CGI-S criteria is provided by data relating to the evaluation of subjective well-being. Indeed, clinical remission evaluated according to CGI-S seems the sole criterion to bear a significant association with subjective well-being, both in terms of the percentage of subjects identified as being in this state and of differences in mean scores between remitters and non-remitters.

Prior to drawing conclusions, several limitations characterizing the present study should be taken into account. First, the sample size of the study was rather limited; second, the study focused solely on chronic outpatients who referred to the centre over a specific period, thus excluding patients who had moved away, refused to continue treatment or no longer needed continuing care. Therefore, the findings emerging from the study should be applied only to chronic patients undergoing long-term treatment. Additionally, as sample heterogeneity is considered one of the main flaws of remission studies [12], the inclusion in the present study of patients affected by both schizophrenia and schizoaffective disorders should be taken into account. However, clinical remission rates observed were consistently similar among patients with schizoaffective and schizophrenic disorders, independent of remission criteria adopted. Moreover, although the proportion of schizoaffective patients found to be in functional remission was higher than among schizophrenics, this difference was not statistically significant. Additionally, rates of functionally remitted patients detected among clinically remitted patients, both schizophrenic and schizoaffective, independent of the remission criteria adopted, were similarly higher. It should be taken into account that the severity criterion alone, without the duration criterion, was used in evaluating remission. Remission studies generally demonstrate how use of the severity criterion alone is associated with higher remission rates [12] compared to use of both severity and duration criteria. However, as pointed out also in a critical review [9], the majority of studies used RSWG criteria only, neglecting duration, as this choice is often more feasible for study design. We may assume that rates of remission found in this study using both RSWG and CGI criteria would be similar, although lower, even when adopting the time component in evaluating remission. Accordingly, we hope to confirm the validity of this hypothesis once data from the 2-year follow-up of the present study become available. At present, we acknowledge that the abovementioned limitation prevents us from drawing any firm conclusions as to the validity of complete remission criteria.

Finally, it should be underlined how raters taking part in this study, due to their expertise with PANSS and other rating scales, may, at times, have evaluated the same patient, thus enhancing familiarity with patients’ symptoms and creating a potential bias toward consensus. Moreover, no structured interview guide, recently reported to improve the accuracy of data obtained, was used in evaluating global clinical impression [32]. However, it should be emphasized that every effort was made to avoid, whenever possible, assessment of patients by the same resident and that a good inter-rater reliability for CGI-SCH evaluations was confirmed before starting the study. Thus, even in the light of the above limitations, the evidence collected may be of relevance in clinical practice.

As expected, the present study confirms the validity of severity remission criteria proposed by the RSWG in determining a better symptomatologic and functional profile, lending further support to the findings of Van Os et al. [10] who reported how the use of standardized remission criteria in schizophrenia “had the potential to improve documentation of clinical status in medical records, by providing an objective measure of illness course and treatment effect that is applicable to routine clinical care”. Moreover, the data obtained in this study indicate how the CGI-Severity criterion may represent an appropriate alternative to RSWG criteria, both in pragmatic studies and in routine practice, due to the ease of application of CGI-SCH in assessing the clinical conditions of patients and their remission status, based upon a rapid assessment of all dimensions of symptomatology in schizophrenia. Moreover, the possibility of a more user-friendly method for use in the evaluation of remission, may contribute towards extending this approach to the routine care system, with the consequent advantage of a more concise, and in our opinion, more useful way of assessing outcomes of therapeutic interventions. However, it should be highlighted that although time-consuming, the use of the entire PANSS, not limited to selected items such as those included in RSWG criteria [4], would seem to be better suited for research purposes, being associated with the best performances in describing remission [33].

## References

[1] Nasrallah HA, Lasser R (2006). Improving patient outcomes in schizophrenia: achieving remission. J Psychopharmacol.

[2] Davidson L, Schmutte T, Dinzeo T, Andres-Hyman R (2008). Remission and recovery in schizophrenia: practitioner and patient perspectives. Schizophr Bull.

[3] Masand P, O’Gorman C, Mandel FS (2011). Clinical Global Impression of Improvement (CGI-I) as a valid proxy measure for remission in schizophrenia. Analyses of ziprasidone clinical study data. Schizophr Res.

[4] Andreasen NC, Carpenter WT, Kane JM, Lasser RA, Marder SR, Weinberger DR (2005). Remission in schizophrenia: proposed criteria and rationale for consensus. Am J Psychiatry.

[5] Kane JM, Crandall DT, Marcus RN, Eudicone J, Pikalov A, Carson WH (2007). Symptomatic remission in schizophrenia patients treated with aripiprazole or haloperidol for up to 52 weeks. Schizophr Res.

[6] Leucht S, Beitinger R, Kissling W (2007). On the concept of remission schizophrenia. Psychopharmacology (Berl).

[7] De Hert M, van Winkel R, Wampers M, Kane J, van Os J, Peuskens J (2007). Remission criteria for schizophrenia: evaluation in a large naturalistic cohort. Schizophr Res.

[8] Peuskens J, Kaufman L, Van Vleymen B (2007). Analysis of resolution criteria in patients with schizophrenia treated with olanzapine for an acute psychotic episode. Schizophr Res.

[9] AlAqeel B, Margolese HC (2012). Remission in schizophrenia: critical and systematical review. Harv Rev Psychiatry.

[10] van Os J, Burns T, Cavallaro R, Leucht S, Peuskens J, Helldin L (2006). Standardized remission criteria in schizophrenia. Acta Psychiatr Scand.

[11] Leucht S, Davis JM, Engel RR, Kissling W, Kane JM (2009). Definition of response and remission in schizophrenia: recommendations for their use and their presentation. Acta Psychiatr Scand Suppl.

[12] Lambert M, Karow A, Leucht S, Schimmelmann BG, Naber D (2010). Remission in schizophrenia: validity, frequency, predictors and patients’ perspectives 5 years later. Dialogues Clin Neurosci.

[13] Carpiniello B, Pinna F, Tusconi M, Zaccheddu E, Fatteri F. Gender differences in remission and recovery of schizophrenic and schizoaffective patients: preliminary results of prospective cohort study. Schizophrenia Research and Treatment 2012, Article ID 576369. doi:10.1155/2012/576369. 10.1155/2012/576369 PMC3420715 22966440

[14] First MB, Spitzer RL, Williams JBW, Gibbon M (1996). Structured Clinical Interview for DSM IV Axis I Disorders-Research Version (SCID-I, Version 2.0).

[15] First MB, Gibbon M, Spitzer RL, Williams JBW, Benjamin L (1996). Structured Clinical Interview for DSM IV Axis II Personality Disorders-Research Version (SCID II, Version 2.0).

[16] Conti L, Dell’Osso L, Cassano GB (1988). Il sistema AMDP. Manuale per la valutazione e la Documentazione della Psicopatologia. Versione Italiana.

[17] Carpiniello B, Baita A, Carta MG, Sitzia R, Macciardi AM, Murgia S (2002). Clinical and psychosocial outcome of patients affected by panic disorder with or without agoraphobia: result of a naturalistic follow-up study. Eur Psychiatry.

[18] Primavera D, Bandecchi C, Lepori T, Sanna L, Nicotra E, Carpiniello B (2012). Does duration of untreated psychosis predict very long term outcome of schizophrenic disorders? Results of a retrospective study. Ann Gen Psychiatry.

[19] Kay SR, Fiszbein A, Opler LA (1987). The positive and negative syndrome scale (PANSS) for schizophrenia. Schizophr Bull.

[20] Haro JM, Kamath SA, Ochoa S, Novick D, Rele K, Fargas A (2003). The Clinical Global Impression-Schizophrenia scale: a simple instrument to measure the diversity of symptoms present in schizophrenia. Acta Psychiatr Scand Suppl.

[21] Keefe RS, Goldberg TE, Harvey PD, Gold JM, Poe MP, Coughenour L (2004). The Brief Assessment of Cognition in Schizophrenia: reliability, sensitivity and comparison with a standardized neurocognitive battery. Schizophr Res.

[22] Anselmetti S, Poletti S, Ermoli E, Bechi M, Cappa S, Venneri A (2008). The Brief Assessment of Cognition in Schizophrenia. Normative data for the Italian population. Neurol Sci.

[23] Folstein MF, Folstein SE, McHugh PR (1975). Mini Mental State. A practical method for grading the cognitive state of patients for the clinician. J Psychiatr Res.

[24] Measso G, Cavarzeran F, Zappalà C, Lebowitz BD, Crook TH, Pirozzolo FJ (1993). The Mini Mental State Examination. Normative study of an Italian random sample. Dev Neuropsychol.

[25] Morosini PL, Magliano L, Brambilla L, Ugolini S, Pioli R (2000). Development, reliability and acceptability of a new version of the DSM-IV Social and Occupational Functioning Assessment Scale (SOFAS) to assess routine social functioning. Acta Psychiatr Scand.

[26] Naber D, Moritz S, Lambert M, Pajonk FG, Holzbach R, Mass R (2001). Improvement of schizophrenic patients’ subjective well-being under atypical antipsychotic drugs. Schizophr Res.

[27] Brissos S, Dias VV, Balanzá-Martinez V, Carita AI, Figueira ML (2011). Symptomatic remission in schizophrenia patients: relationship with social functioning, quality of life, and neurocognitive performance. Schizophr Res.

[28] Karow A, Moritz S, Lambert M, Schöttle D, Naber D (2012). EGOFORS Initiative. Remitted but still impaired? Symptomatic versus functional remission in patients with schizophrenia. Eur Psychiatry.

[29] Mosolov SN, Potapov AV, Ushakov UV (2012). Remission in schizophrenia; results of cross-sectional with 6 months follow up period and 1-year observational therapeutic studies in an outpatients population. Ann Gen Psychiatry.

[30] Shrivastava A, Johnston M, Shah N, Thakar M, Stitt L (2011). Persistent cognitive dysfunction despite clinical improvement in schizophrenia: a 10-year follow-up. J Psychiatr Pract.

[31] Goordwod P, Burns T, Juckel J, Rossi A, San L, Hargarter L (2013). Psychiatrists’ perceptions of the clinical importance, assessment and management of patient functioning in schizophrenia in Europe, the Middle East and Africa. Ann Gen Psychiatry.

[32] Targum SD, Houser C, Northcutt J, Little JA, Cutler AJ, Walling DP (2013). A structured interview guide for global impression: increasing reliability and scoring accuracy for CNS trials. Ann Gen Psychiatry.

[33] Pinna F, Tusconi M, Bosia M, Cavallaro R, Carpiniello B, Cagliari Recovery Group Study (2013). Criteria for symptom remission revisited: a study of patients affected by schizophrenia and schizoaffective disorders. BMC Psychiatry.

